# Determination of oxidative stress level and some antioxidant activities in refractory epilepsy patients

**DOI:** 10.1038/s41598-024-57224-6

**Published:** 2024-03-20

**Authors:** Abdullah Yilgor, Canan Demir

**Affiliations:** 1 Department of Neurology, Faculty of Medicine, Van YuzuncuYil University, 65200 Van, Turkey; 2 Vocational School of Health Services, Van, Turkey

**Keywords:** Catalase, Malondialdehyde, Refractory epilepsy, Superoxide dismutase, Development of the nervous system, Neuroimmunology

## Abstract

The aim of this study was to determine the levels of superoxide dismutase (SOD), catalase (CAT), reduced glutathione (GSH) and malondialdehyde (MDA) in patients with refractory epilepsy. Serum superoxide dismutase (SOD), catalase (CAT), reduced glutathione (GSH) and malondialdehyde (MDA) levels were determined using the spectrophotometer method. Refractory epilepsy patients’ serum superoxide dismutase (SOD), catalase (CAT), reduced glutathione (GSH) and malondialdehyde (MDA) levels were statistically significant compared to the healthy control group (*p* < 0.05). In conclusion, superoxide dismutase (SOD), catalase (CAT), reduced glutathione (GSH) and malondialdehyde (MDA) levels may play an important role in the etiopathogenesis of refractory epilepsy. This study was the first to investigate some parameters in refractory epilepsy disease.

## Introduction

Epilepsy is known as a serious disease of the nervous system. Loss of consciousness usually occurs in epilepsy patients^1–4^.Epilepsy is a very commonly observed disease globally. This disease causes physical, psychosocial and economic problems in society^5^. Recently, studies of epilepsy have intensified. Despite the effective doses of antiepileptics used in the treatment of epilepsy, seizure control cannot be achieved, because the disease has become very resistant to drugs. In epilepsy, seizures can occur continuously^6,7^. Refractory epilepsy is an important disease in which seizure control cannot be achieved. Different drugs and doses are used to fight against this disease. However, seizure recurrence continues in epileptic patients^8,9^. The main method for diagnosis, treatment and follow-up of epilepsy is age, gender, seizure types, genomic studies, technological advances, electroencephalography (EEG) and imaging^10^. Drugs used in the treatment of epilepsy provide seizure control, but do not have an effect on epileptogenesis^11^.

Free radicals are unpaired electron pairs. The main free radicals are compounds such as hydroxy (OH^−^), peroxy (ROO.), and superoxide (O2^−^) radical. When free radicals are formed, they become stable. Also, these radicals enter the structure of cells and damage them. As a result of this damage, various diseases may occur. It has been reported that free oxygen radicals play an important role in the etiopathogenesis of various diseases. It has been determined that free oxygen radical damage occurs in diseases such as bladder disease, prostate cancer, sepsis, myocardial infarction, stroke, perinatal hypoxic brain injury, glomerulonephritis, uveitis, various cancers and arthritis in laboratory, clinical and experiments^12–18^. However, antioxidants protect the cell against these radicals^17–20^.

Malondialdehyde (MDA) is the end product of lipid peroxidation. MDA occurs in the formation of free oxygen radicals or when arachidonic acid occurs. MDA is known as an indicator of oxidative stress. When free radicals increase, MDA level rise as well. In the literature, it has been found that MDA, a marker of oxidative stress, has a higher level in patients with epilepsy^21^. Another study with similar results reported that MDA levels are higher in epilepsy patients compared to healthy individuals^22^.

Catalase (CAT) is an enzyme in the oxidoreductase group. It has four heme groups. It is in the structure of CAT hemoprotein. CAT converts hydrogen peroxide (H_2_O_2_) into water and oxygen. It is one of the most powerful antioxidants^23^. It has been reported that CAT activity in patients with adult epilepsy have decreased activity compared to healthy individuals in the patient group^24^. Literature studies have determined that CAT activity in epilepsy patients have decreased activity compared to healthy individuals^22^.

Serum superoxide dismutase (SOD) is a very resistant antioxidant in the cell. It is also a free radical scavenger. It converts the superoxide radical into hydrogen peroxide and oxygen^25^. In a study on epilepsy, it has been found that SOD activity increased in the patient group^26^. Conversely, in another study, SOD activity has been found to be lower in epilepsy patients than in healthy individuals^22^.

Reduced glutathione (GSH) removes reactive oxygen molecules from the cell. It is synthesized in the liver. At the same time, it has 3 tripeptides in its structure. There are two types of GSH, endogenous and exogenous^27^. In a study, it has been reported that the GSH level in epileptic patients did not change compared to healthy individuals^28^. In another study, it was determined that the GSH level increased in epileptic patients^29^.

In this study, we provide a brief discussion on the role of oxidative stress and some antioxidant activities in the pathophysiology of refractory epilepsy. The role of neuroprotectants in the therapeutic strategy to prevent or treat epilepsy is also discussed. In this study, our first aim was to investigate the relationship between oxidative stress and in refractory epilepsy. This study was the first to investigate some parameters in refractory epilepsy disease. Additionally, the second aim of this study is to determine the levels of superoxide dismutase (SOD), catalase (CAT), reduced glutathione (GSH) and malondialdehyde (MDA) in resistant epilepsy patients who have not been studied before.

## Materıals and methods

Patients who experience more than 1 seizure a month and show a tolerance to medication were accepted as refractory epileptic^30^.

In this study, blood samples were taken from 35 healthy individuals and 35 patients diagnosed with refractory epilepsy. 2 ml of blood was taken from the antecubital venous vein and added to a biochemistry tube.

### Method

A total of 70 people, 35 of whom were diagnosed with refractory epilepsy and 35 healthy controls, who applied to the Neurology Department of Van Yüzüncü Yıl University, were included in the study. The age range of the patient group was 33.09 ± 13.4. The age range of the control group was 34 ± 13. Most of the subjects included in this study were patients whose seizures could not be fully controlled despite using effective doses of 1st generation and 2nd generation antiepileptic drugs^31^. All of the patients were examined in detail and EEG and IMR of the patients were taken. In addition, demographic characteristics of the patients, frequency of seizures, seizure type, duration of disease and medications they used were recorded (Table 1). Patients with hypertension, diabetes, rheumatoid arthritis, chronic neurological disease, a history of smoking, alcohol and those using any other non-prescription drugs were not included in the study. Patients with refractory epilepsy who had inflammatory conditions and those who took antioxidants as supplements or used medication were also excluded from the study. We assured that all methods were carried out in accordance with Scientific Report’s relevant guidelines and regulations and also informed consent was obtained from all subjects and/or their legal guardians.

**Table 1:** Demographic data of the patient and control groups.

	Patient	Control
n	35	35
Age (mean ± S.D)	33.09 ± 13.4	34 ± 13 (*p* = 0.142)
Gender		
Male (n,%)	14, 40%	24, 68.6%
Female (n,%)	21, 60%	11, 31.4%
Patients		
EEG None (n, %)	21, 60%	
Pathological (n, %)	14, 40%	
MR None (n, %)	23, 65.7%	
Pathological (n, %)	12, 34.3%	
Seizurefrequency (n, %)	1 times 3 months: 2, 5.7%	
	1 times month: 1, 2.8%	
	2 times month: 24, 68.6%	
	2–3 times month:1, 2.9%	
	3 times month: 3, 8.6%	
	3–4 times month: 3, 8.6%	
	3 times month: 1, 2.8%	
Seizure type (n, %)	Focal: 9, 25.8%	
	Focal-jk: 18, 51.4%	
	JK: 8, 22.8%	
Drugs (n, %)	Lev: 7, 20%	
	Other: 28, 80%	

In the study, biochemical parameters were determined with serum samples. Local ethics committee approval was obtained from Van Yüzüncü Yıl University Medical Faculty Hospital before blood samples were taken (decision number:03 and date:10.02.2021). Laboratory and imaging results of the patients were recorded. For this study, 2 ml of blood was taken from the antecubital venous vein and added to a biochemistry tube. Afterwards, the blood samples were centrifuged at 5.000 rpm for 10 min and the serums were separated from the plasma. From the serum samples, superoxide dismutase (SOD) activity, catalase (CAT) activity, reduced glutathione (GSH) levels and malondialdehyde (MDA) levels were determined.

### Biochemical investigations

#### Determination of superoxide dismutase (SOD) activity

SOD activity was measured in serum samples in both the patient and healthy control groups. SOD activity was determined in accordance with the method proposed by Gunduz et al^18^. The SOD analysis was measured as thus: Two tubes were taken, one was serum and the other tube was blank. 1.425 µl of reagent was added to the blank and sample tubes. 50 µl of serum was added to the sample tube. In the blank tube, no serum was added. Then, while 100 µl of bidistilled water was added to the blank tube, it was not added to the sample tube. 25 µl of xanthine oxidase was added to both the blank and the sample tube. This mixture was incubated at 25 °C for 20 min. Finally, the blank and sample tubes were scanned vis a vis bidistilled water at 560 nm.

% Inhibition = [(blank OD–Sample OD)/blank OD] × 100

#### Determination of reduced glutathione (GSH) level

GSH level was measured in serum samples in both refractory epilepsy and healthy control groups. The GSH level was determined in accordance with the Tietz method^32^. 800 µl of phosphate buffer was added to 200 µl of serum. Initial absorbance (OD1) at 412 nm was measured. 100 µl of Ellman’s reagent was added to the same tube and then the second absorbance (OD2) was measured.

#### Calculation

Glutathione concentration was calculated in mmol/g protein unit.

C/1000 = (OD2 − OD1) / 13,600 × E1 × 5/2  × ½

13,600: Molar extinction coefficient of yellow color formed during the interaction of GSH and 5,5-dithio-bis-(2-nitrobenzoic acid) (DTNB).

E1: If a band with a width greater than 6 nm is used, a derivative extrusion coefficient is used for correcting both light path and bandwidth differences. The width of the tape we used was 2 nm. It was taken as E1 = 1 in the calculations.

1000: conversion coefficient to mmol.

C: mmol/glutathione (mg/dl).

#### Determination of catalase (CAT) activity

CAT activity was measured in serum samples in both refractory epilepsy and healthy control groups. In this study, CAT activity was determined in accordance with the Aeibi method. Firstly, 1.4 ml of 30 mM H_2_O_2_ was placed in the blank tube and 0.1 ml of phosphate buffer was added to it. 1.4 ml of 30 mM H_2_O_2_ was placed in the sample tube and 0.1 ml of sample was added to it and the tubes were vortexed. The absorbance values were then measured twice at 240 nm at 30 s intervals using the spectrophotometric method^33^.

Activity account:

Activity = (2.3/ΔX) × [(log A1/log A2)].

ΔX: 30 s.

2.3: 1 mmol optical density of H_2_O_2_ in 1 cm light path.

#### Determination of malondialdehyde (MDA) level

MDA level was measured according to the Bird and Draper method^34^. 200 ml of serum was added to the sample tube, then 800 ml of phosphate buffer, 25 ml of butylated hydroxy toluene (BHT) solution and 500 ml of 30% tricarboxylic acid (TCA) were added to it. The tubes were mixed by vortexing and kept on ice for 2 h. It was then centrifuged at 2000 rpm for 15 min. 1 ml of the obtained supernatant was taken and transferred to another tube. Then 75 ml of ethylenediamine tetraacetic acid (EDTA) and 250 ml of thiobarbutiric acid (TBA) were added to this mixture. The tubes were mixed by vortex again and kept in a hot water bath for 15 min. The tubes were then brought to room temperature. Absorbance values were read in the spectrophotometer at 532 nm.

Calculation of malondialdehyde level:

C = F × 6.41 × A.

C: Concentration, F: Dilution factor, A: Absorbance.

### Statistical analysis

The descriptive statistics for the features mentioned were expressed as mean, standard deviation, median, min and max for continuous variables, numbers and percentages for categorical variables. The population of the study consists of a total of 200 refractory epilepsy patients who applied to Van Yüzüncü Yıl University Dursun Odabaşı Medical Center, Department of Neurology in the last 6 months. The number of samples was determined by the Universal Population Volume Known Sampling Calculation Formula (n = Nz^2^pq/d^2^(N − 1) + z^2^pq). According to the literature review; It was determined that the "effect size" value was 0.15. According to this; Taking the Primary Type Error as 5% (Z = 1.96), the Power of the Test as 80% and the effect size as 0.15 units; the minimum sample size was calculated as n = 35.2. Thus, considering the study process, the study was planned with a total of 70 volunteers, including 35 patients diagnosed with resistant epilepsy and 35 healthy controls. The T-Test was used in the comparison of binary groups providing the condition of normal distribution. The statistics of the Mann–Whitney U-test was used when the condition of normal distribution was not provided. The statistical significance level was taken as *p* < 0.05 and the SPSS statistical software pack version 19.0 (SPSS Inc, Chicago, III, USA) was used for analyses.

### Ethical approval

The authors confirm that they have read the Journal’s position on issues involved in ethical publication and affirm that this report is consistent with those guidelines.

## Results

**Figure 1:**
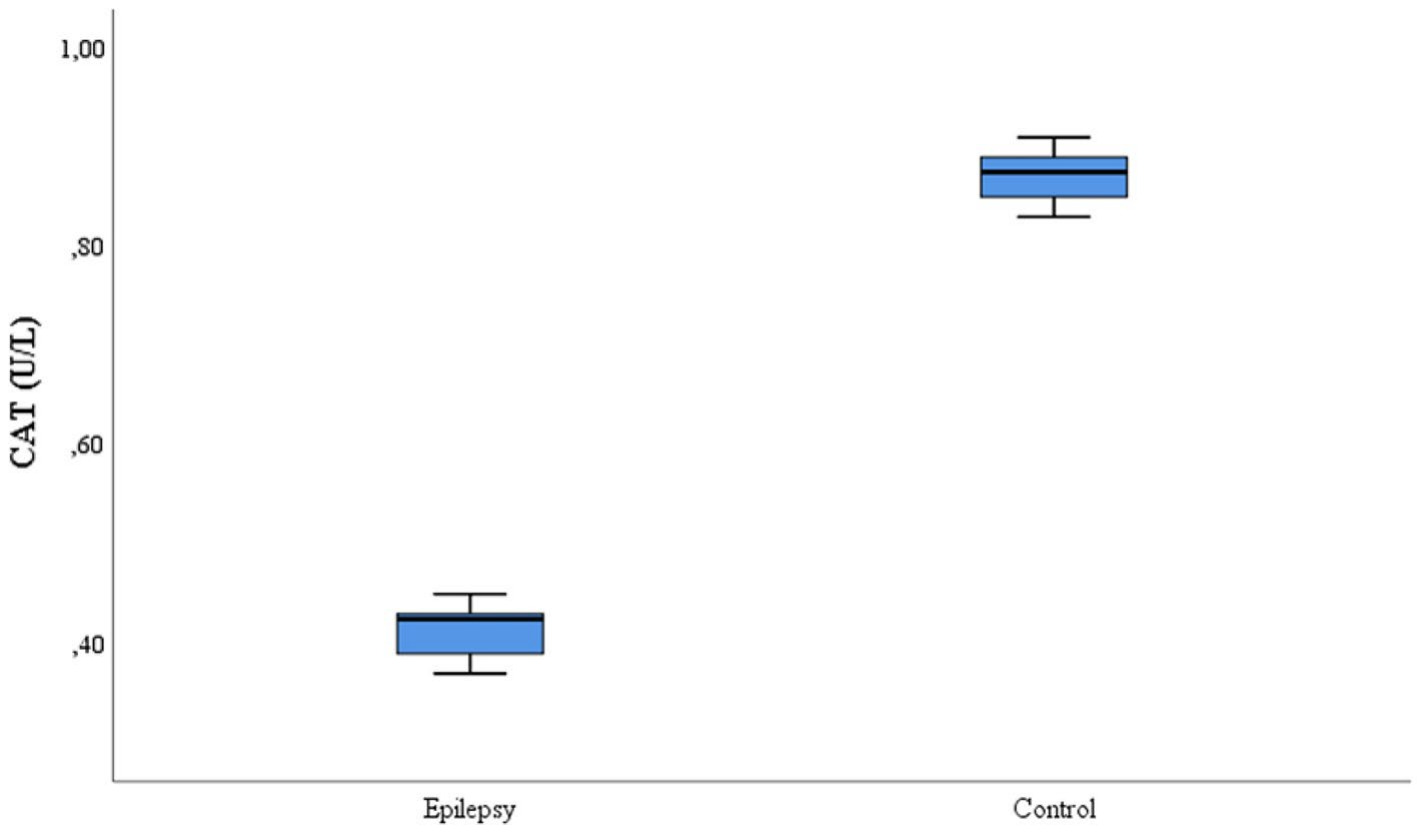
CAT activities between the refractory epilepsy and the healthy control group.

**Figure 2:**
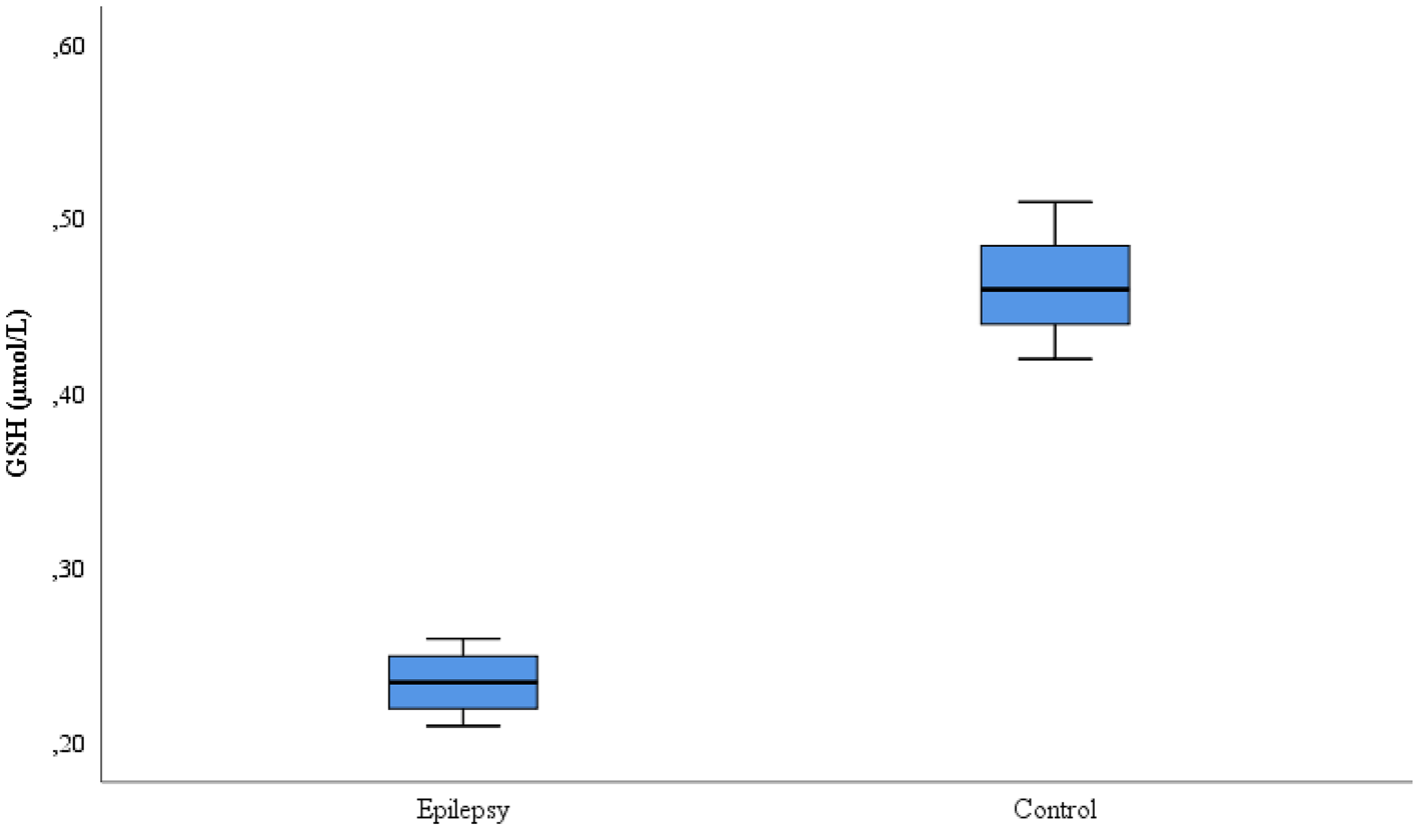
GSH levels between the refractory epilepsy and the healthy control group.

**Figure 3:**
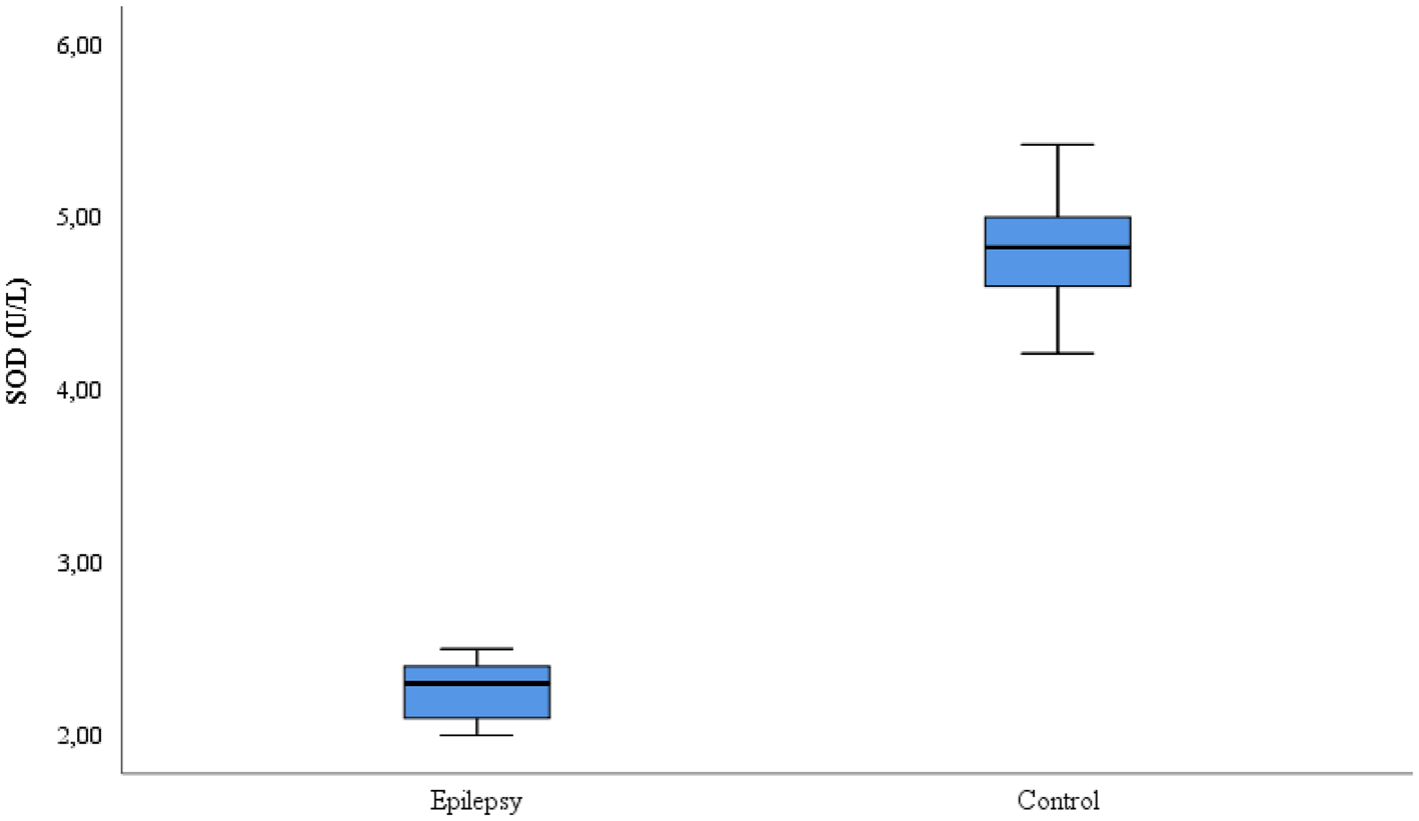
SOD activities between the refractory epilepsy and the healthy control group.

**Figure 4:**
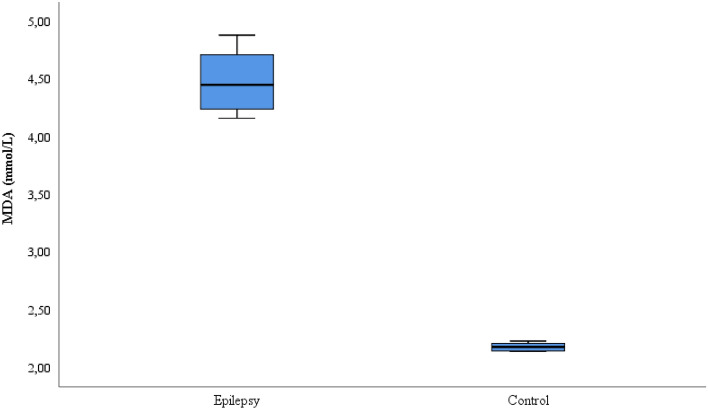
MDA levels between the refractory epilepsy and the healthy control group.

This study included 35 patients with refractory epilepsy and 35 control subjects. The age, gender, seizure frequency, seizure type, EEG, IMR and medication information of the patient and control groups is given in Table 1. The CAT, SOD, GSH, and MDA values comparison for the patient and control groups is shown on Table 2. While the CAT, GSH and SOD values were lower in the refractory epilepsy group compared to the control group, the MDA values were higher in the refractory epilepsy group (Figs. 1, 2, 3 and 4).

**Table 2:** Biochemical parameters between refractory epilepsy and healthy control group.

	Refractory epilepsy					Control					*p*
	Mean	S.D	Median	25%	75%	Mean	S.D	Median	25%	75%	
CAT (U/L)	0.42	± 0.03	0.43	0.39	0.43	0.87	± 0.02	0.88	0.85	0.89	** < 0.001**
GSH (µmol/L)	0.24	± 0.02	0.24	0.22	0.25	0.46	± 0.03	0.46	0.44	0.49	** < 0.001**
SOD (U/L)	2.28	± 0.17	2.30	2.1	2.4	4.84	± 0.33	4.82	4.6	5.0	** < 0.001**
MDA(mmol/L)	4.48	± 0.25	4.45	4.24	4.71	2.15	± 0.08	2.18	2.15	2.21	** < 0.001**

## Discussion

Epilepsy is a neurological disorder. It is a very common disease. Refractory epilepsy is a much more serious disease. In these patients, seizure control is never achieved^8,9^. Epilepsy patients face very serious risks in daily life. The quality of life of these patients declines significantly. Likewise, these patients have serious psychological problems^35^. Epilepsy is more common in men than in women in general. Seizures in this disease are often more common in the elderly people and younger children. Controversy continues about the causes of this disease. The research of the etiopathonenesis of this disease are still ongoing^36^. Surgery has recently become important in the treatment of refractory epilepsy^8^.

Free radicals cause malondialdehyde levels to rise which causes oxidative stress. Free radicals are unpaired atoms. It is known that free radicals are responsible for diseases such as atherosclerosis, diabetes, epilepsy, inflammatory diseases and cancer. Oxidative stress occurs when the balance between oxidant and antioxidants is damaged^37,38^. Oxidative stress leads to metabolic problems in the membrane lipids, DNA, RNA, and the increased risk of seizures and recurrent seizures^39^. There is a relationship between the brain and oxidative stress. Thus, when the input of the oxygen to the metabolism is intense, the brain signals. As a result of increased oxidative stress, cerebral diseases such as brain damage or epilepsy occur^39,40^.

It has been determined that diseases such as Alzheimer's, Parkinson’s, and multiple sclerosis (MS) are neurodegenerative diseases and that there is an increase in the level of oxidative stress in patients with these diseases. Free oxygen radicals or oxidative stress cause structural changes in the cell. Studies have reported that oxidative stress is effective in depression and different psychological stress diseases^41–43^.

MDA is a marker reflecting oxidative stress. MDA also shows lipid peroxidation. The autocatalytic chain reaction involved in lipid peroxidation is very dangerous to the cell and initiates cellular damage. In a study, it was found that the MDA level increased in epilepsy patients compared to the healthy control group^21^. In another study, the MDA levels were found to be higher in individuals with normal epilepsy^44^. In this study, MDA levels were found to be significantly higher in patients with refractory epilepsy when compared to healthy individuals (*p* < 0.05). (Fig. 4). According to these results, high MDA levels in refractory epilepsy patients may be another indicator of increased oxidative stress. The results show us that brain damage has occurred. Because of the high amount of lipids in the brain, intense oxygen consumption and increased oxidative metabolism show us that the brain is sensitive to oxidative stress. This may be the beginning of refractory epilepsy disease. In addition, MDA may cause structural differences in the platelet mechanism. Thus, prostaglandin release and cerebral blood flow decrease in the metabolism. In conclusion, increased oxidative stress may play an important role in the pathogenesis of refractory epilepsy. MDA was investigated for the first time in refractory epilepsy. In addition, this study will contribute to the literature.

Antioxidants fight free radicals and also reduce the effects of free radicals. SOD, CAT, GST, GR (glutathione reductase), GPx (glutathione peroxidase) and GSH are the main antioxidants. Antioxidant enzymes are protective type enzymes that increase their activity under oxidative stress conditions^17–20^. Antioxidants are required as compensatory mechanisms of oxidative stress. Otherwise, oxidative stress may cause cellular damage. Strong antioxidants are needed to conteract this. Thanks to the antioxidants found in the body, it is possible to protect from cellular damage.

SOD is a very powerful antioxidant enzyme. SOD ensures the excretion of many toxic and harmful compounds from the body. It also removes free radical products from the cell. In a study, it was reported that the SOD level decreased significantly in patients with migraine^17^. In another study, it was reported that SOD activity decreased in bladder cancer patients compared to healthy control groups^45^. In a presented study, it was reported that the SOD activity decreased in epilepsy patients^26^. In the literature, it has been determined that SOD activity is decreased in epilepsy patients compared to healthy individuals.^22^ In this study, it was observed that SOD enzyme activity decreased significantly in refractory epilepsy patients compared to healthy individuals (*p* < 0.05) (Fig. 3). The cause of this is likely the high accumulation of lipid compounds in the brain in patients with refractory epilepsy. Oxidative stress rises and can also trigger refractory epilepsy. Thus, antioxidant agents, especially SOD activity, begin to decrease in the body. SOD activity in refractory epilepsy was studied for the first time. The role of antioxidants in the treatment of refractory epilepsy should be researched in more detail. This study will greatly contribute to the the current literature in these respects.

CAT is one of the most important antioxidant agents. It is a leading compound in the removal of hydrogen peroxide, which is toxic in the cell. It converts hydrogen peroxide into water and molecular oxygen in metabolism. At the same time, CAT prevents cellular damage^18.^ . It has been reported that CAT activity is decreased in young epilepsy patients compared to healthy individuals^24^. In another study, CAT activity has been found to be lower in epilepsy patients^22^. In this study, the serum CAT activity of refractory epilepsy patients was statistically significant compared to the healthy control group (*p* < 0.05), and it was also found to be low in patients. (Fig. 1). This study is the first to reflect CAT activity in refractory epilepsy. It is certain that this study will contribute to the literature.

GSH level protects the cell against reactive oxygen molecules. However, it is a very powerful antioxidant as well. Because GSH protects the cell from all kinds of threats against oxidative stress, it has been reported that cellular damage may occur in GSH deficient patients. The GSH level is higher in patients with prostate cancer and bladder disease^16,17,46^. In experimental rat models, it has been determined that the seizures in animals did not recur for a long time with the administration of some antioxidant agents in animals with epilepsy^47^. In a study on patients with epilepsy, it has been reported that there was no significant difference in the GSH level when compared to healthy individuals^28^. In another study in the literature, it was reported that the GSH level increased in epilepsy patients^29^. In this study, the GSH level decreased significantly in the patient group when compared to the healthy control group (*p* < 0.05) (Fig. 2). This is likely due to an increase in reactive oxygen radicals or oxidative stress in the body. In such a case, damage to the brain may occur which may accelerate the epilepsy process. This study is the first to investigate the level of GSH in refractory epilepsy. This study will contribute to the literature.

In general, low SOD and CAT and high MDA levels were found in epilepsy patients in the literature. But, in some studies, preoperative SOD activity in epileptic patients did not change compared to post-operative patients. Also, in the same study, CAT enzyme activity before and after surgery was not found to be significant compared to the healthy control group^48^. A study by Dalton et al^49^. is the first to determine that severe brain damage in a rat model of epilepsy is in part due to oxidative stress. In a reported study, further research on the possible role of oxidative stress in epilepsy and refractory epilepsy was recommended^50^. This study differs from other studies in the literature, as it is the first study in which parameters such as SOD, CAT, GSH and MDA are measured in the refractory epilepsy disease.

There is evidence that neuronal overstimulation and oxidative damage caused by excessive free radical production may play a role in the onset and progression of epilepsy. Understanding the role of oxidative stress in epileptogenesis is important for determining appropriate treatment strategies. Neuroprotectant or antioxidant compounds may exert positive effects when associated with antiepileptic drugs (AEDs). The role of neuroprotectants in the therapeutic strategy to prevent or treat epilepsy is also discussed. Although some mysteries need to be clarified in the future, antioxidant compounds are useful neuroprotective agents in ameliorating brain damage in patients with refractory epilepsy. As a result, it is important that some antioxidant substances should be included in the treatment protocol for refractory epilepsy. Increased SOD, CAT and GSH levels can be used as a good antioxidant agent against refractory epilepsy disease. Also, MDA may be a precursor marker in identifying the refractory epilepsy disease.

The risk of seizures may increase in refractory epilepsy as a result of the reduction of the antioxidant defense system with the formation of active oxygen metabolites. In such a situation, oxidative stress may play an important role in the mechanism of neuronal death due to epilepsy. Ultimately, the occurrence of epileptic seizures may be the cause and consequence of reactive oxygen radicals. Thus, oxidative phosphorylation in mitochondria can generate large numbers of oxygen radicals in the nervous system. In such cases, it is necessary to reduce the excess oxygen radicals in the nervous system. For this, high antioxidant systems are needed to keep the body immunity strong.

As a result, it is important that some antioxidant substances should be included in the treatment protocol for refractory epilepsy. Increased SOD, CAT and GSH levels can be used as a good antioxidant agent against refractory epilepsy disease. Also, MDA may be a precursor marker in identifying the refractory epilepsy disease. This study was the first to investigate some parameters in refractory epilepsy disease

## Data Availability

The datasets used and/or analyzed during the current study can be provided by the corresponding author upon reasonable request, either by providing it in a supplementary file or by depositing it in a public repository and providing the details on how to access it.
